# Microplastics and human health: unraveling the toxicological pathways and implications for public health

**DOI:** 10.3389/fpubh.2025.1567200

**Published:** 2025-06-18

**Authors:** Xu Zhang, Chunhong Yu, Peng Wang, Chunping Yang

**Affiliations:** ^1^Department of Otorhinolaryngology Head and Neck Surgery, The Second Affiliated Hospital, Jiangxi Medical College, Nanchang University, Nanchang, Jiangxi, China; ^2^Department of Hepatobiliary Medicine, Fuzong Clinical Medical College of Fujian Medical University, Fuzhou, Fujian, China; ^3^Department of Otolaryngology-Head and Neck Surgery, Jiangdu People’s Hospital Affiliated to Yangzhou University, Yangzhou, Jiangsu, China

**Keywords:** microplastics, human health, cellular toxicity, environmental pollutants, systemic health risk

## Abstract

The increasing prevalence of microplastics (MPs) in the environment has raised urgent concerns regarding their implications for human health. This comprehensive review integrates recent findings on the sources, classification, and pathways of MPs into the human body, highlighting their potential cellular toxicity and systemic health risks. We discuss the mechanisms by which MPs may induce inflammatory responses, oxidative stress, and cellular damage, thereby contributing to various diseases. Notably, we examine the synergistic effects of MPs in conjunction with other environmental pollutants, which may amplify their adverse health outcomes. This synthesis of current research underscores the critical need for multidisciplinary approaches to investigate the multifaceted interactions between MPs and human health, ultimately guiding future studies and informing public health strategies to mitigate exposure and associated risks.

## 1 Introduction

Plastics have gained widespread global usage due to their durability, affordability, and various other advantages. However, despite the extensive consumption of plastic materials, only about 20% of plastic waste is recycled or incinerated, leaving a significant portion abandoned in landfills or dispersed throughout the natural environment (121). The generation of global plastic waste is rapidly escalating in tandem with increased plastic production. According to data from Plastics Europe,^1^ it is projected that by 2050, global plastic waste could reach approximately 2.4 billion tons, more than six times the volume produced in 2020. Due to their resistance to degradation, plastics can persist in the environment for decades or even centuries, breaking down into smaller fragments through processes such as physical abrasion, chemical reactions, and biological degradation. Microplastics (MPs) have emerged as a rapidly expanding category of pollutants, raising significant concerns in both environmental and health contexts due to their toxicological effects.

MPs are defined as water-insoluble synthetic solid particles or polymer matrices derived from both primary and secondary sources, typically characterized as plastic particles with dimensions less than 5 mm (1). They are categorized into primary MPs—those directly released into the environment through human activities, such as plastic particles found in personal care products like cleansers and cosmetics—and secondary MPs, which result from the degradation of larger plastic items such as ropes, clothing, and packaging (2). While plastics exhibit environmental persistence, once they enter ecosystems, they undergo chemical weathering, photodegradation, biodegradation, and mechanical forces that compromise their structural integrity. This process leads to fragmentation into particles ranging from micrometers to nanometers in size (3). Humans can be exposed to MPs present in the air through inhalation or dermal contact. In aquatic and soil environments, MPs can accumulate pollutants and enter the food chain via drinking water and agricultural products, potentially leading to biomagnification at higher trophic levels. This accumulation raises significant concerns regarding human health (4).

Given their pervasive nature, microplastic pollution poses a considerable global challenge. It is essential to continually address the origins of these tiny particles and assess their potential impacts on human health. Although numerous review articles have focused on the distribution of MPs in aquatic ecosystems such as oceans and surface waters, research concerning their implications for human populations remains limited, with conclusive evidence regarding health risks still lacking. In this context, we present a review of recent advancements in understanding the toxicity of MPs. This includes an examination of their definition, classification, sources, pathways into the human body, mechanisms of cytotoxicity, health risks associated with human systems, and interactions with other pollutants. Unlike previous reviews predominantly focused on the environmental distribution of MPs, this study advances the field by elucidating their molecular targets in human systems and dissecting toxicological mechanisms through a multidimensional lens. We systematically synthesize exposure pathways across atmospheric, aquatic, and terrestrial matrices while integrating emerging clinical evidence. By overcoming fragmented approaches in existing literature, this review establishes a cohesive theoretical framework linking mechanistic insights to intervention strategies, thereby offering novel perspectives for mitigating MPs-associated health risks and aims to establish a foundation for further exploration in this critical area of research.

## 2 MPs in the environment

### 2.1 Definition of MPs

The persistence of plastics in the environment poses significant challenges, as they can remain intact for decades or even centuries. Over time, these materials fragment into smaller particles due to processes such as physical abrasion, chemical reactions, and biodegradation. In 2004, Thompson et al. identified plastic particles approximately 20 μm in size along British coastlines and in marine environments, leading to the introduction of the term “microplastics” (MPs) (5). MPs are now defined as plastic fragments or particles with diameters less than 5 mm, and their widespread presence in daily life results in unavoidable human exposure (6).

MPs can manifest in various forms, with their properties influenced by their origins and the environmental conditions they encounter. Factors such as residence time, the initial structure of primary plastics, and degradation processes—including photodegradation, mechanical wear, and biological contamination—play crucial roles in shaping these particles (7). Generally, fibrous, granular, and fragmented MPs arise from larger plastic items through mechanisms like photodegradation and mechanical action (8). Microplastic particles are typically spherical and range from a few micrometers (μm) to several millimeters (mm) in size, with most diameters falling between 1 mm and a few mm. In contrast, microplastic fibers are longer and narrower than other types of MPs, measuring from 10 μm to several millimeters in length. Due to their small size and varied forms, MPs can easily contaminate food sources, increasing ingestion risk. Furthermore, they tend to accumulate within the human body more readily and are more challenging to eliminate.

### 2.2 Classification of MPs

**Figure 1:**
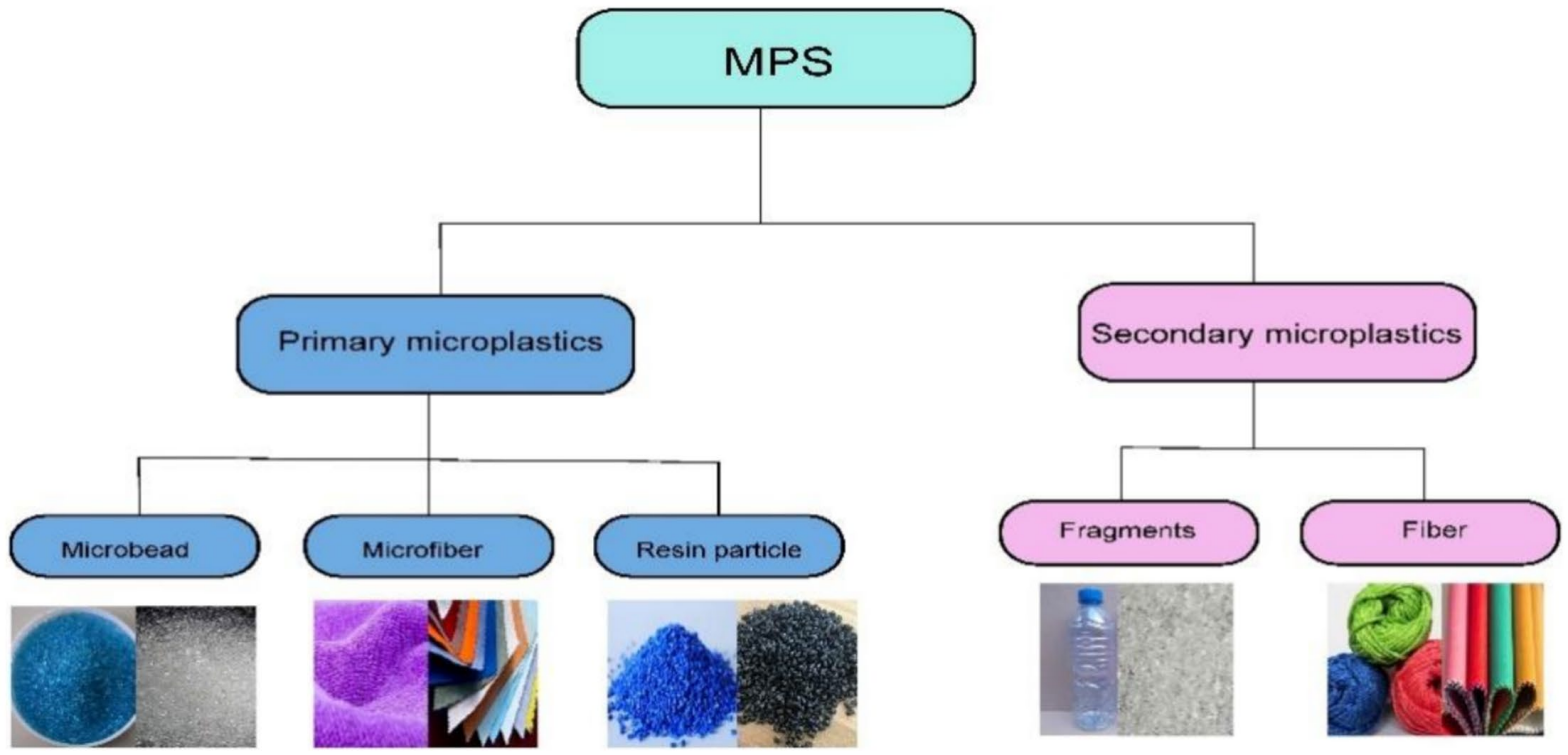
Classification and sources of MPs.

Based on the sources of MPs, they can be divided into two major categories: primary MPs and secondary MPs (2). Figure 1 and Table 1 provides an overview of the classification and sources of MPs.

**Table 1:** The types and sources of MPs.

Classification of microplastic	Type of microplastic	Source of microplastic	Reference
Primary microplastics	Microbread	Personal care and cosmetic items such as exfoliating scrubs and toothpaste.	Nawalage et al. (18)
	Microfiber	Textiles like synthetic clothing, carpets, and home furnishings. Personal care products, including cigarette filters, wet wipes, and face masks.	Athey and Erdle (12)
	Resin particle	Plastic product	Mortensen et al. (4)
Secondary microplastics	Fragments	The breakdown of larger plastic.	Emenike et al. (14)
	Fiber	Textiles, including garments, ropes, and fishing nets.	Emenike et al. (14)

Primary MPs are defined as small plastic particles that are either manufactured at a microscale or produced as by-products during the production process. These particles are specifically designed for various applications, including their use as injection molding powders, abrasive grains, or resin particles (9). Additionally, primary MPs can arise from the wear and tear of larger plastic items throughout their lifecycle, such as the abrasion of tires during driving or the shedding of synthetic fibers during laundering (10). Common forms of primary MPs include microbeads, microfibers, and resin pellets.

Microbeads are tiny plastic spheres, typically measuring less than 5 mm in diameter, that are often found in personal care and cosmetic items such as exfoliating scrubs and toothpaste. Their primary role in these products is to act as abrasives or to enhance texture. Due to their small size, microbeads can easily enter aquatic systems and often evade filtration processes at wastewater treatment facilities (11). Microfibers are fine plastic filaments that originate from textiles like synthetic clothing, carpets, and home furnishings. These fibers can be released at various stages of a textile’s lifecycle, including during production, usage, washing, and even post-treatment. Microfibers are also present in personal care products, including cigarette filters, wet wipes, and face masks (12). Resin pellets serve as raw materials for plastic product manufacturing and can pose environmental risks to aquatic ecosystems if mishandled or accidentally released during production, transportation, or processing (4). These primary MPs have the potential to adsorb and transport hazardous chemicals, thereby increasing their ecological impact.

Secondary MPs are small plastic particles generated from the degradation and fragmentation of larger plastic products such as bags, bottles and Wrapping materials. This breakdown occurs due to mechanical forces like waves and abrasion, chemical reactions, and exposure to ultraviolet radiation from sunlight (13). Given the frequent release of larger plastic items into the environment, secondary MPs constitute the primary components of microplastic pollution. The predominant forms of secondary MPs include fragments and fibers (14). Plastic fragments are unevenly shaped remnants produced through the breakdown of larger plastic items, whereas fibers are slender strands originating from textiles, including garments, ropes, and fishing nets.

### 2.3 Origin of MPs

**Figure 2:**
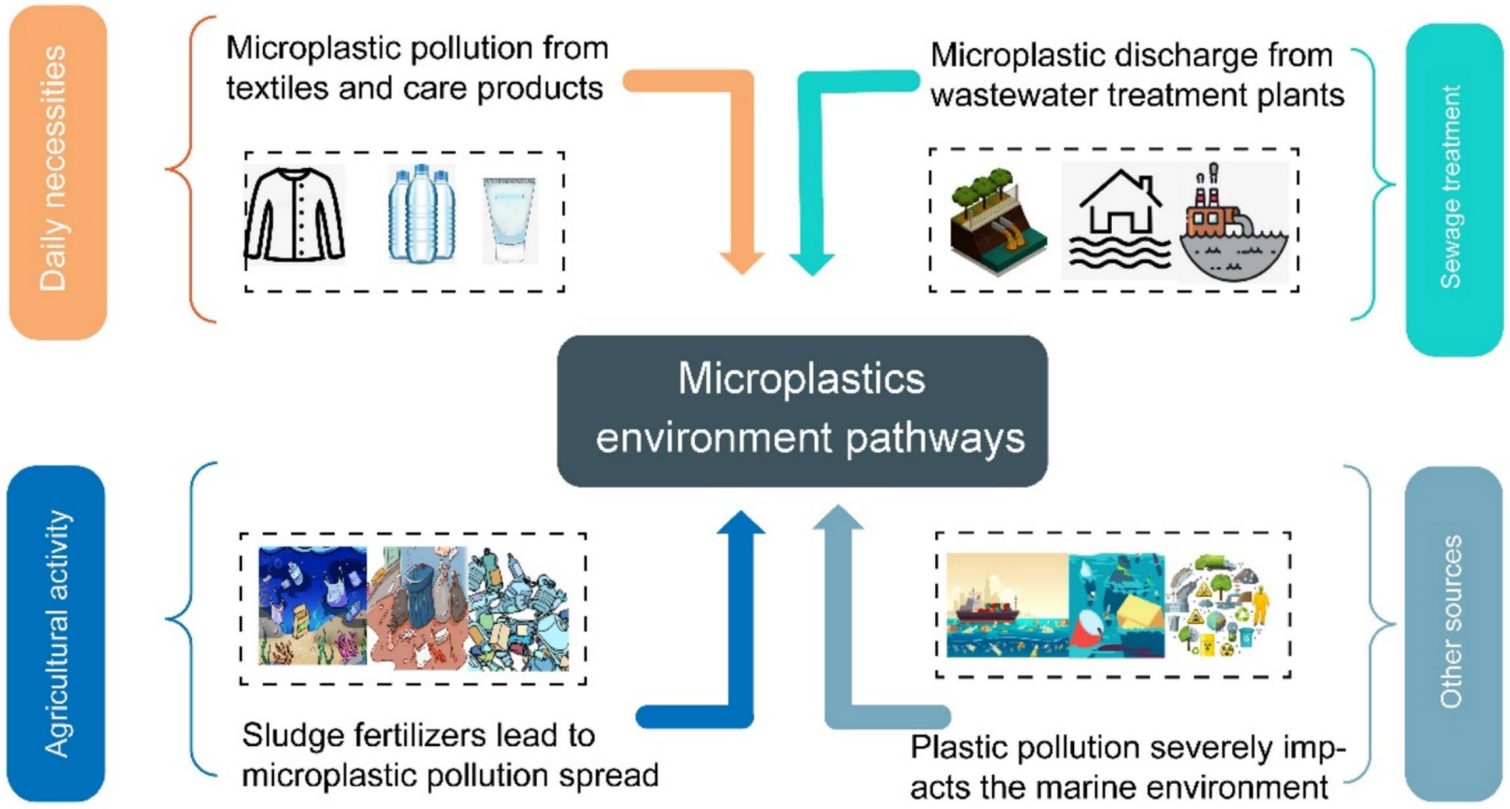
Sources of various MPs.

The classification of microplastics is closely intertwined with their environmental origins. MPs have numerous sources. Major sources include the use of everyday products (such as detergents, cosmetics), agricultural activities, wastewater discharge, and more (Figure 2).

#### 2.3.1 Daily supplies

Textiles, especially those made from synthetic materials like nylon and polyester, as well as natural fibers such as wool, significantly contribute to microplastic pollution (15). The laundering of these textile products subjects them to mechanical and chemical stresses, resulting in the release of tiny microfibers. Due to their diminutive size, these microfibers are often unable to be effectively filtered out by wastewater treatment facilities, leading to their eventual discharge into aquatic ecosystems (16). Studies have shown that on average, washing a single piece of clothing can release between 1,900 and 1 million microfibers, while washing textiles made primarily of polyester can result in the release of over 6 million fibers. These findings highlight the significant contribution of laundry activities to microplastic pollution in the environment (17).

In personal care products, primary MPs are predominantly found, including microbeads, microfibers, and other forms. These products typically include soaps, shampoos, conditioners, body washes, cosmetics, and skin care products. The exfoliating microbeads are found in care products such as facial cleansers and toothpaste (18). The ingredients labeled in personal care products, such as polyethylene, polypropylene, polyethylene terephthalate, and polymethyl methacrylate, may be primary components of microplastics. When used in everyday life, the primary MPs they generate can bypass wastewater treatment plants and enter aquatic environments through wastewater. Simultaneously, they can also be ingested by aquatic organisms. Microbeads are often added to products as exfoliants or abrasives, and they are intentionally manufactured as small plastic particles. When these items are laundered, the microbeads may be released into aquatic environments, thereby exacerbating microplastic pollution. The ingestion of these MPs by aquatic life can lead to a range of ecological issues, including potential harm to the health of the organisms and the transfer of pollutants up the food chain (19). Studies have shown that a single use of toothpaste during brushing can release approximately 4,000 microbeads (17). A survey conducted across various supermarkets in China revealed that 7.1% of facial cleansers and 2.2% of body washes were found to contain microplastics, with polyethylene identified as the primary material comprising these MPs (20).

#### 2.3.2 Wastewater treatment plant

Wastewater treatment facilities are primarily engineered to eliminate organic matter, nutrients, and various pollutants from wastewater; however, they are not particularly effective in removing MPs (21). Treated wastewater can enter aquatic environments through multiple routes, including the direct discharge of effluent, overflow during storm events, and the use of sewage sludge as fertilizer (22). Furthermore, there is a notable connection between wastewater treatment plants and the textiles and personal care products that contribute to MP pollution. Addressing this relationship could provide a viable strategy for mitigating the presence of MPs. A study conducted on two wastewater treatment facilities in Turkey found that influent water contained between 1 million and 6.5 million microplastic particles daily, while the effluent water ranged from 220,000 and 1.5 million particles per day. The research identified seven distinct types of polymers, with polyester comprising the majority of those detected (23).

#### 2.3.3 Rural activity

Agricultural land fertilizers often originate from sludge produced by industrial wastewater treatment, aiming to recycle organic matter and provide nutrients (24). Consequently, this can lead to a considerable buildup of MPs in agricultural lands, from which these particles can subsequently enter aquatic ecosystems through multiple pathways, including rainfall, leaching, and irrigation practices (25). A study evaluated MPs in 124 organic compost samples, including those derived from single feedstocks (e.g., livestock/poultry manure, crop straw, solid waste) and composite materials. Results revealed significant variations in MPs abundance, with solid waste compost exhibiting the highest concentration (6,615 items/kg), whereas crop straw compost contained the lowest (1,500 items/kg). Annual MPs input to agricultural soils via compost application was estimated at 6.96 × 10^7^–1.88 × 10^8^ items/ha (26). Analysis of soil MPs in China’s Weishan Irrigation District demonstrated significant variations in MP abundance across land-use types, with mean concentrations of 900, 512,615, and 633 items/kg detected in vegetable fields, croplands, orchards, and woodlands, respectively. MPs were predominantly characterized by small particle sizes (0.2–1 mm, >78% of total), blue/purple or transparent coloration (>60%), and film-, fiber-, or fragment-like morphologies (>78%). Polyethylene (>36%) was the most prevalent polymer type. Notably, organic fertilizers were identified as a major contributor to MP contamination (27).

#### 2.3.4 Other sources

Additionally, plastics enter water systems through casual disposal and fishing gear (28). These materials undergo degradation when exposed to sunlight, resulting in the formation of various microplastic fragments. Notably, beach litter constitutes roughly 80% of the plastic waste present in ocean environments. Furthermore, practices such as recreational pursuits, and unregulated fishing, combined with the growing trend of human migration to coastal areas, suggest that the introduction of plastic waste into marine ecosystems is expected to increase in the future. The wide distribution of microplastics in the environment enables them to enter the human body through a variety of ways and affect human cells and organs.

## 3 The mechanisms of pollutant adsorption on MPs

MPs are widely distributed in the environment and their unique surface properties make them ideal carriers for various harmful microorganisms, forming “contaminant-microplastics complexes” with potential transmission risks (2). Current studies confirm that microplastic surfaces exhibit significant adsorption capacity for various environmental contaminants, including organic pollutants, heavy metals, and pathogenic microorganisms (29, 30). Due to their substantial surface area and hydrophobic properties, MPs serve as significant carriers for contaminants. Additionally, the mechanisms of contaminant adsorption onto MPs also involve electrostatic repulsion and attraction, pore blockage, and site competition (31). For instance, studies have demonstrated that arsenic can adsorb onto microplastics through non-covalent bonding and electrostatic interactions (32, 33), resulting in enhanced arsenic accumulation and synergistic toxic effects (34). In a study, the inhibitory effect of organic matter on activated carbon fibers was reported. The research demonstrated that there was no significant adsorption of organic matter itself, while the concentration of organic matter markedly increased (35). These findings suggest a competitive adsorption between organic matter and perfluorooctanoic acid, as well as pore blockage of activated carbon fibers by organic matter.

## 4 Pathways of MPs into human cells

MPs primarily enter the bodies of organisms, including humans, through consumption, inhalation, and skin contact. Traces of MPs have been detected in feces, meconium, placentas, lung tissue, breast milk, saliva, blood, facial tissue, liver, kidneys, and the colon (36).

### 4.1 Inhalation by respiration

**Figure 3:**
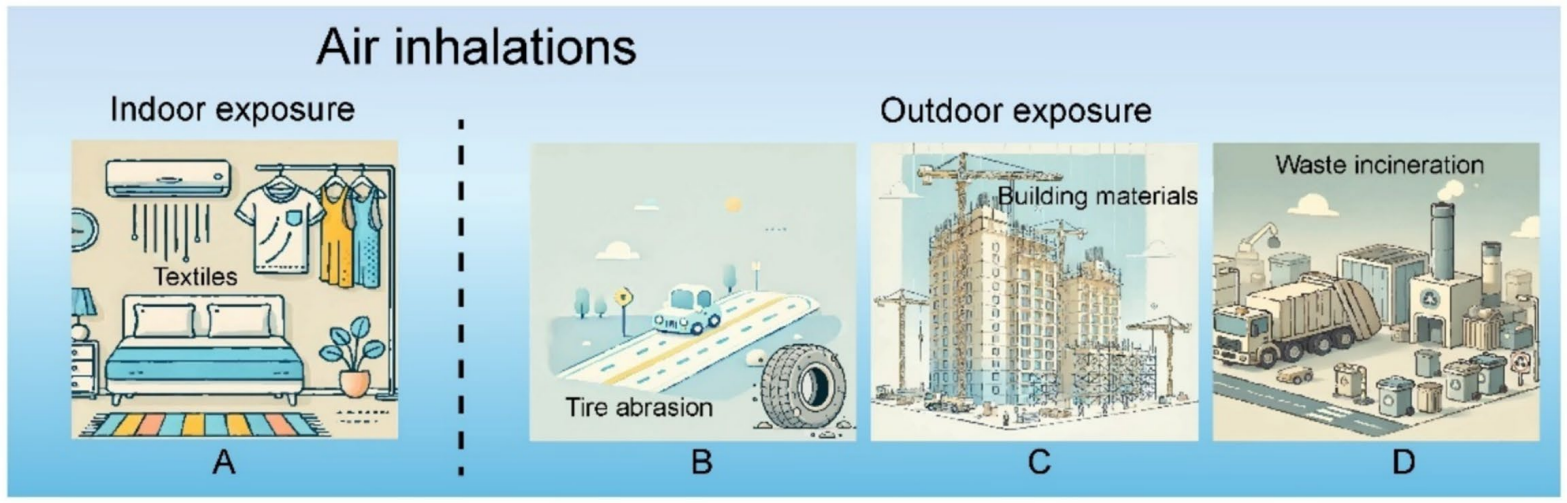
MPs inhaled from indoor and outdoor air environments.

Inhalation via the respiratory system represents a significant route for the introduction of MPs into the human body (Figure 3), with prior research validating their presence in ambient air (37). Notably, MPs measuring less than 5 μm in length and under 3 μm in diameter are particularly susceptible to inhalation, as they can often evade the mucociliary clearance mechanisms of the upper respiratory tract. Once inhaled, these MPs can disseminate throughout the body via the upper digestive system, ultimately accumulating in various organs (38).

Tire wear, construction materials, and waste incineration are primary contributors to the presence of MPs in outdoor environments (39). Research indicates that approximately 3–7% of particulate matter measuring less than 2.5 μm in the atmosphere originates from tire wear, with an average annual emission rate of 0.81 kg of tire dust per person (40). Additionally, polyethylene and polystyrene are frequently utilized as insulating and molding materials in construction, and the expansion of large-scale architectural projects has led to increased MP emissions (41). A study on emissions from incineration reveals that between 1.9 and 565 microplastic items per kilogram of Municipal Solid Waste (MSW) accumulate in the incinerator’s bottom ash, with the incineration process facilitating their release into the atmosphere (42, 43).

Factors like wind direction, altitude, and population density significantly influence the concentration of MPs in outdoor settings. One study found that urban areas exhibit a higher atmospheric concentration of MPs (13.9 items/m^3^) compared to rural regions (1.5 items/m^3^) (44). Another investigation in German cities reported no detectable plastic concentrations in less populated areas (0 particles/m^3^) (45). Furthermore, this research highlighted elevated MP concentrations near the ground, peaking at a height of 1.7 m—nearly three times greater than those measured at 33 and 80 m (45). Wind direction plays a crucial role in the dispersion of plastic particles; the distribution of MPs is significantly correlated with prevailing wind patterns (45). Consequently, atmospheric MPs tend to accumulate in urban locales, from which they can be transported over considerable distances by wind.

For students and white-collar workers, a significant portion of their time is spent indoors. A recent study found that the concentration of indoor MPs ranges between 1.0 and 60.0 fibers/m^3^, its main component is polysulfated mucopolysaccharides (a type of MPs). Textiles are a common source of polysulfated mucopolysaccharides in indoor environments, primarily composed of fiber particles such as polyamide, acrylic, and polyester (46, 47). Fiber MPs are the most common type of MPs found in indoor environments. Research has shown that the level of indoor MPs (ranging from 1.0 to 60.0 fibers/m^3^) is significantly higher than the level of outdoor MPs (ranging from 0.3 to 1.5 fibers/m^3^) (46). The concentration of polyethylene terephthalate (PET) is between 1,550 and 120,000 mg per kilogram (mg/kg) indoors, which is higher than the range of 212–9,020 mg/kg outdoors (48).

The direction and strength of the airflow generated by air conditioners can affect the migration of polysulfated mucopolysaccharides (a type of MPs) into indoor spaces. A study conducted in student dormitories on airflow testing showed that turbulent airflow can lead to the resuspension of MPs, MPs particles are prone to depositing in open food and drinking water indoors. Moreover, activities such as walking, closing doors, or engaging in some indoor exercises can potentially lead to the resuspension of MPs within indoor spaces (49).

### 4.2 Oral ingestion

The intake of seafood, such as fish, shrimp, and shellfish, constitutes a major route for MPs to infiltrate the human body. Studies indicate that MPs are commonly found in the tissues and organs of marine species, thereby enabling their transmission to humans via the food chain (50). A survey of the Chinese fishery market revealed that various types of MPs—such as fibers, fragments, and particles—were present in all samples of commercially harvested bivalve shellfish, with particles averaging below 250 μm accounting for 33–84% of the total MPs detected (51). In Indonesia, Rochman et al. discovered that 55% of fish samples were contaminated with MPs, with the majority being less than 500 μm in size and mainly consisting of polyethylene and polypropylene (52).

Bottled drinking water is another significant source of MPs, originating from both the plastic bottles and caps. Over time, degradation of these materials can introduce MPs into the water supply (53). Research indicates that consuming tap water leads to an estimated intake of 4,000 MPs annually, while bottled water consumption can result in an additional intake of approximately 90,000 MPs per year (54). A study by Sherri A. Mason and colleagues analyzed 259 bottled water products across nine countries and found that 93% contained MPs. The average concentration of microplastic particles larger than 100 μm was recorded at 10.4 per liter, with fragments and fibers being the predominant forms. Polypropylene was identified as the most common polymer, comprising 54% of the total (55). In Germany, all bottled water samples tested positive for MPs, with 80% of particles ranging from 5 to 20 mm. Recyclable plastic bottles exhibited the highest average microplastic content at 118 particles per liter, primarily composed of polyester (PET) and polypropylene (PP), while single-use bottles contained an average of 14 particles per liter and beverage cartons had 11 particles per liter, with polyethylene being the common material (56).

Moreover, MPs are found in a variety of food sources such as drinking water, beverages, milk, canned goods, sugar, and salt (57). Studies confirm that low-nutrition organisms are more susceptible to environmental MP contamination. Recent research employing two complementary analytical techniques identified that 32% of MPs smaller than 20 μm were present in drinking water samples. This underscores the need for high-performance filtration systems to enhance purification processes (58, 59). Additionally, a study indicated that the risk of ingesting plastic from mussels is lower compared to exposure to fibers from dust during meals, estimating an annual range of ingestion between 13,731 and 68,415 particles per person (60). Yinan Li and colleagues have also reported that commonly consumed beverages worldwide—including beer, tea, and honey—contain MPs in various forms such as particles, fragments, and fibers. These contaminants are primarily attributed to raw materials as well as environmental exposure during processing and packaging (61).

### 4.3 Skin contact and dermal absorption

MPs can enter the human body and other organisms through dermal exposure to a range of topical products, such as cosmetics, body washes, topical medications, and surgical or prosthetic devices. They can also be absorbed through occupational interactions in both indoor and outdoor environments. Under typical circumstances, MPs do not breach the subcutaneous barrier; however, their capacity to penetrate this barrier is primarily determined by their size and chemical characteristics. Particles measuring less than 100 nm are more likely to penetrate the skin due to their smaller size and increased surface reactivity. Furthermore, larger particles may be absorbed via hair follicles, sweat glands, or damaged areas of the skin (62, 63).

## 5 Mechanism of cytotoxicity induced by MPs

**Figure 4:**
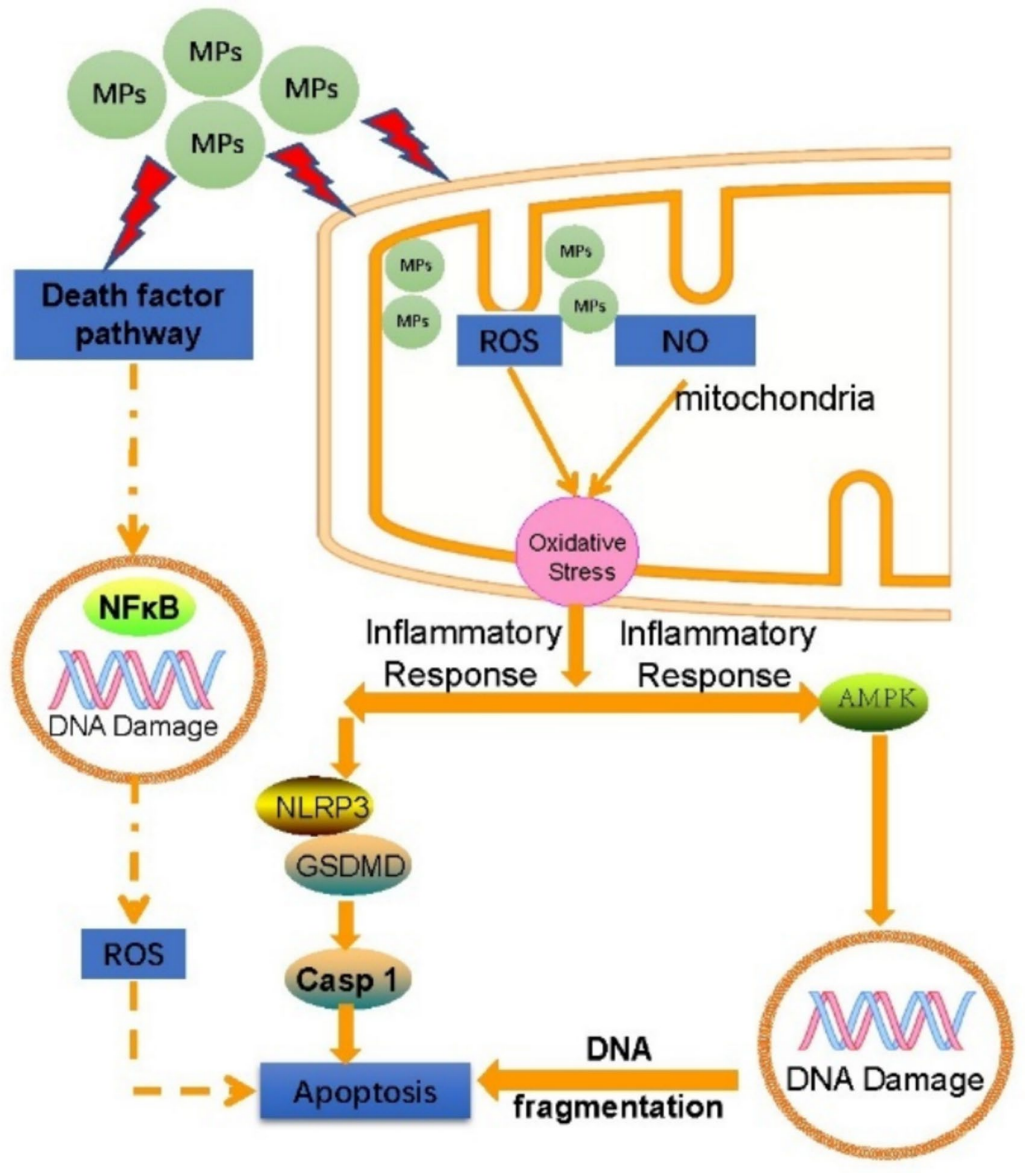
Toxicity mechanisms of MPs on cells and organs.

Upon entering mammalian cells, MPs can engage in interactions that elicit a range of cellular responses, including Reactive oxygen species (ROS) stress, Inflammatory responses, Cellular apoptosis, and genomic instability characterized by DNA damage (Figure 4). Once MPs enter mammalian cells, they are enclosed by lysosomes for degradation before being released into the cytoplasm. This release may lead to mitochondrial dysfunction, which in turn increases the production of ROS. Elevated ROS levels can undermine the cell’s antioxidant defense systems, resulting in protein oxidation, lipid peroxidation, and DNA damage (64).

### 5.1 Oxidative stress

Mitochondria serve as the primary source of reactive oxygen species (ROS), and oxidative stress arises from an imbalance between ROS production and the capacity of the antioxidant defense system. MPs can disrupt mitochondrial membrane potential and hinder mitochondrial energy production, contributing to this imbalance (65, 66). Research by Hao Wu et al. demonstrated that polystyrene MPs can induce DNA damage, cell cycle arrest, and necrotic apoptosis in mouse ovarian granulosa cells by enhancing ROS production (67). Furthermore, MPs have been shown to diminish the activity of antioxidant enzymes *in vitro*, thereby triggering oxidative stress. For instance, polystyrene MPs can lead to hepatocyte apoptosis and alter glycolytic flux through ROS-mediated calcium overload (68, 69).

### 5.2 Inflammatory reaction

MPs can stimulate the release of cytokines such as IL-6, IL-8, and IL-10, leading to immune-mediated inflammatory responses that may ultimately result in necrosis of cellular or tissue structures (70). In a murine study, MPs were shown to enhance the expression of inflammatory proteins cPLA2 and COX-1, which subsequently caused renal damage in the mice (71). Research involving chicken cardiomyocytes revealed that MPs could alter the NF-κB-NLRP3-GSDMD and AMPK-PGC-1α signaling pathways due to ROS overload, thereby inducing oxidative stress, pyroptosis in cardiomyocytes, inflammation, and impairments in mitochondrial function and energy metabolism (72). Furthermore, a study conducted on rats suggested that MPs could induce both pyroptosis and apoptosis in ovarian granulosa cells through the NLRP3/Caspase-1 signaling pathway, potentially triggered by ROS (73).

### 5.3 Apoptosis

Research has demonstrated that exposure to MPs in zebrafish models leads to a significant upregulation of apoptosis-related genes, including p53, gadd45ba, and casp3b, ultimately resulting in the degradation of gill tissue structure (74). Similarly, another study reported that MPs markedly activated NF-κB, pro-inflammatory cytokines, and apoptosis markers in human microglial cells as well as in mouse brains. This activation included increased levels of BAX, cleavage of PARP, and the activation of caspases 3 and 8, culminating in the apoptosis of microglial cells in both species (75). Additionally, Siwen Li and colleagues found that MPs can induce hepatocyte apoptosis through calcium overload driven by reactive oxygen species (ROS) (68).

### 5.4 Gene damage

In a study measuring various stress indices in zebrafish exposed to MPs, including Lipid oxidative damage, DNA lesions, autophagic process, caspase activation, metabolite alterations, and changes in ventricular contraction frequency and force—parameters that correlate with the fish’s swimming speed—it was determined that DNA damage was the most pronounced effect observed (76). Research involving human peripheral blood lymphocytes revealed that exposure to MPs significantly elevated the frequency of micronuclei (MN), nuclear bridge formation (NPB), and nuclear bud formation (NBUD). Notably, even at lower concentrations, MPs contributed to increased genomic instability. The mechanical interactions between MPs and cells, along with the release of additives from MPs, are potential mechanisms underlying this enhanced genomic instability (77). A further investigation revealed that contact with MPs can lead to DNA lesions in both the mitochondria and nucleus, which results in the movement of double-stranded DNA fragments into the cytoplasm. This event activates the DNA-sensing adaptor protein STING, subsequently initiating the cGAS/STING signaling pathway. This cascade of reactions promotes the translocation of NFκB into the nucleus, where it enhances the expression of pro-inflammatory cytokines, ultimately contributing to liver fibrosis (78). Additionally, a study on fish demonstrated that even at low concentrations, nanoparticles (NPs) can penetrate the nucleus and cause DNA damage, as evidenced by an increased incidence of abnormal erythrocyte nuclei (79). The above cytotoxic mechanisms can further cause multiple organ dysfunction such as nervous system and cardiovascular system.

## 6 Health risk of MPs to different organ

**Figure 5:**
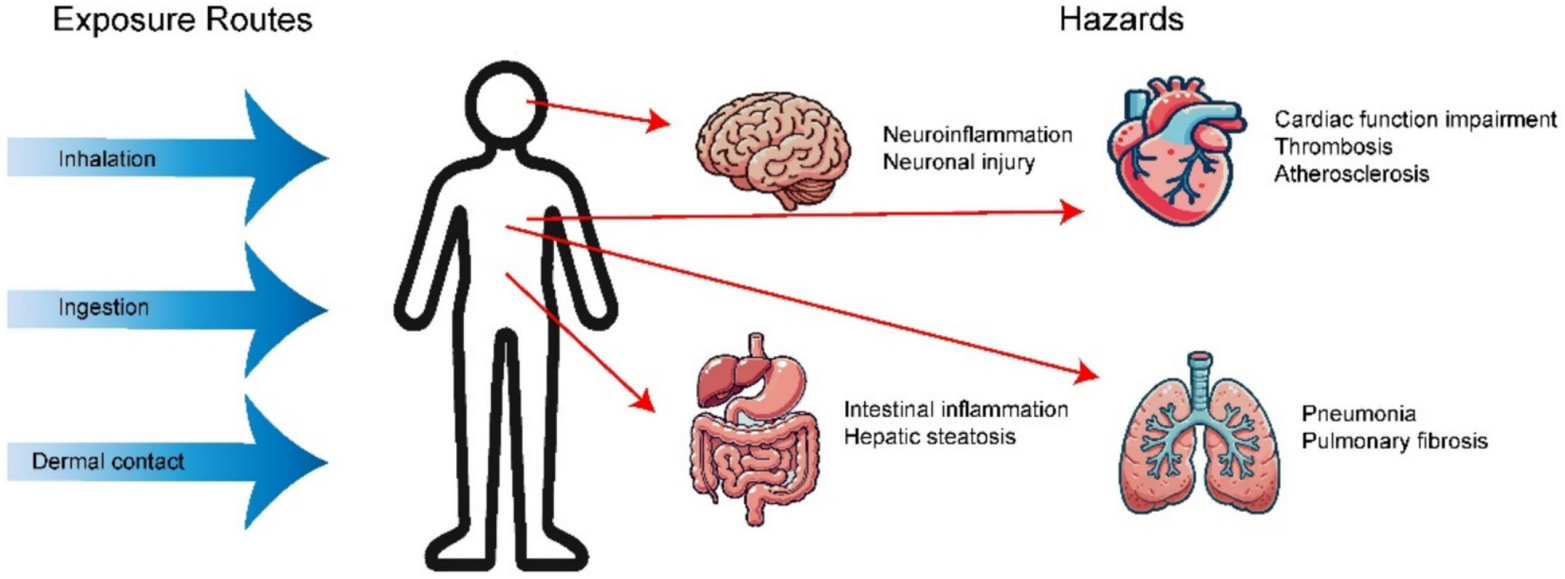
The impact of MPs on different human systems.

Research suggests that exposure to these microscopic plastic particles can lead to a range of detrimental effects on human health. (Figure 5 and Table 2).

**Table 2:** The impact of MPs on different human systems.

Disease system	Type of microplastic	Research model	Exposure route	Toxicity mechanism	Results	Reference
Nervous system	Polystyrene MPs (PS-MPs)	Mouse	Oral Ingestion	Inflammatory response, apoptosis	Polystyrene MPs (PS-MPs) pose a potential risk in microglial immune activation, leading to apoptosis in mouse and human microglia.	Kwon et al. (75)
	Polystyrene MPs (PS-MPs)	Mouse	Oral Ingestion	Gene damage, inflammatory response	Mice exposed to PS-MPs show altered expression of neuron activity-dependent genes and synaptic proteins, increased hippocampal neuroinflammation, indicating adverse effects on learning and memory.	Lee et al. (80)
	Polystyrene MPs (PS-MPs)	Mouse	Oral Ingestion	Oxidative stress, inflammatory response	Mice exposed to PS-MPs exhibit impaired learning and memory through oxidative stress induction and acetylcholine level reduction.	Wang et al. (81, 82)
	Polystyrene MPs (PS-MPs)	Zebrafish	Oral Ingestion	Oxidative stress	Long-term exposure to PS-MPs causes circadian rhythm movement disorder and significant behavioral changes.	Sarasamma et al. (83)
	Polystyrene MPs (PS-MPs)	Mouse	Oral Ingestion	Oxidative stress, inflammation, gene damage	PS-MPs may induce Parkinson-like neurodegenerative changes in mice by reducing ATP levels and downregulating ATP-related genes and protein expression.	Liang et al. (84)
	Polystyrene MPs (PS-MPs)	Mouse	Oral Ingestion	Inflammatory response, apoptosis	MPs exposure reduces mixed neuronal cell viability in mice, with increased levels of apoptosis marker cleaved caspase-3.	Jung et al. (114)
	Polystyrene MPs (PS-MPs)	Mouse	Oral Ingestion	Oxidative stress, inflammation, apoptosis	PS-MPs are internalized into cells. This process triggers ROS production, NF-κB activation, and TNF-α secretion, ultimately leading to necroptosis in hCMEC/D3 cells. It also induces microglial activation and neuronal damage in the brain.	Shan et al. (85)
	Polystyrene MPs (PS-MPs)	Mouse	Oral Ingestion	Oxidative stress, inflammation	Mice in the exposure group show blood–brain barrier disruption, hippocampal inflammation, and cognitive and memory deficits.	Jin et al. (86)
	Polystyrene MPs (PS-MPs)	*C. elegans*	Injection	Inflammatory response	Dopaminergic neuron degeneration, motor dysfunction, and α-synuclein aggregate accumulation observed.	Jeong et al. (115)
	Polystyrene MPs (PS-MPs)	Mouse	Oral Ingestion	Inflammatory response, apoptosis	MPs exacerbate neuroinflammation through microglial pyroptosis, promoting AD cognitive impairment.	Wang et al. (87)
Cardiovascular system	Polystyrene MPs (PS-MPs)	Human pluripotent stem cell-derived 3D cardiac organoid (CO) model	Injection	Oxidative stress, inflammation, apoptosis, gene damage	COs exhibit increased oxidative stress, inflammatory response, and apoptosis, with significant changes in hypertrophy-related genes (MYH7B/ANP/BNP/COL1A1) and cardiac-specific markers (MYL2/MYL4/CX43).	Zhou et al. (90)
	Polystyrene MPs (PS-MPs)	Zebrafish Embryo	Injection	Oxidative stress, inflammation, gene damage	MPs inhibit intestinal blood vessel formation in transgenic zebrafish embryos, potentially interfering with cardiovascular development. Resulting vascular endothelial dysfunction and hypercoagulability further accelerate thrombosis.	Sun et al. (91)
	Polystyrene MPs (PS-MPs)	Mouse	Oral Ingestion	Inflammatory response	MPs aggravate atherosclerosis by upregulating macrophage receptors and activating phagocytosis of M1 macrophages in the aorta.	Wang et al. (92)
	Polystyrene MPs (PS-MPs)	Rat	Oral Ingestion	Oxidative stress, inflammation, apoptosis	MPs have been shown to induce NLRP3 inflammasome activation in mouse heart tissue, activating caspase-1 dependent signaling pathways triggered by oxidative stress-induced inflammation.	Wei et al. (116)
	Polystyrene MPs (PS-MPs)	Rat	Oral Ingestion	Oxidative stress, apoptosis	MPs induce oxidative stress, activating the fibrosis-associated Wnt/β-catenin signaling pathway, leading to cardiac fibrosis and cardiomyocyte apoptosis.	Li et al. (94, 95)
	Polystyrene MPs (PS-MPs)	Chicken	Oral Ingestion	Oxidative stress, inflammation, apoptosis	PS-MPs trigger apoptosis through the NF-κB-NLRP3-GSDMD axis and exacerbate myocardial inflammation. They also induce mitochondrial dysfunction through AMPK-PGC-1α pathway inhibition, causing oxidative stress and mitochondrial dysfunction.	Zhang et al. (72)
	Polystyrene MPs (PS-MPs)	Mouse endothelial and immune cells	Injection	Inflammatory response	MPs enhance cytokine and adhesion molecule expression in the aorta, inducing vascular inflammation.	Vlacil et al. (117)
	Polystyrene MPs (PS-MPs)	Human and Mouse Macrophages	Injection	Oxidative stress	MPs stimulate the accumulation of lipid droplets in macrophages, leading to foam cell formation and promoting atherosclerosis.	Florance et al. (118)
Digestive system	Polystyrene MPs (PS-MPs)	Mouse	Oral Ingestion	Inflammatory response	Mice fed with MPs show significant inflammation in the intestines (colon and duodenum) and higher expression of TLR4, AP-1, and IRF5.	Li et al. (94, 95)
	Polyethylene MPs (PE-MPs)	Caco-2 cells	Injection	Inflammatory response	Key pathways associated with NF-κB, MAPK signaling, cytokine-cytokine receptor interaction, and toll-like receptor strongly affected by PS-MBs.	Wu et al. (96)
	Polyethylene MPs (PE-MPs)	Triple culture transwell model of Caco-2/HT29-MTX-E12/THP-1	Injection	Inflammatory response, gene damage	PE-MPs particles induce DNA damage and inflammatory effects in the model.	Busch et al. (97)
	Polystyrene MPs (PS-MPs)	Mouse	Injection	Inflammatory response	Accumulation of MPs in liver tissue stimulates inflammation, inducing insulin resistance.	Huang et al. (99)
	Polystyrene MPs (PS-MPs)	Zebrafish	Injection	Oxidative stress	PS-MPs also induce significant increases in superoxide dismutase and catalase activity, indicating oxidative stress following MPs treatment.	Lu et al. (100)
	Polystyrene MPs (PS-MPs)	Diabetic Mouse	Injection	Inflammatory response	PS-MPs exposure significantly increases liver tissue damage, inflammatory effects, metabolic disturbances in the liver, and gut microbiota composition changes in diabetic mice.	Liu et al. (102)
Respiratory system	Polystyrene MPs (PS-MPs)	Rat	Inhalation	Inflammatory response, gene damage	MPs upregulate circRNAs and lncRNAs, promoting increased pro-inflammatory cytokines IL-6, TNF-α, and IL-1β, leading to lung inflammation.	Fan et al. (105)
	Polystyrene MPs (PS-MPs)	Human Lung Epithelial BEAS-2B Cells	Injection	Oxidative stress, inflammatory response	PS-MPs induce cytotoxicity and inflammation in BEAS-2B cells by generating reactive oxygen species.	Dong et al. (32, 33)
	Polystyrene MPs (PS-MPs)	Human Alveolar Epithelial Cells, HPAEpiC	Injection	Oxidative stress, inflammation, apoptosis	PS-MPs disrupt redox balance, induce inflammation, and trigger apoptosis pathways.	Yang et al. (42, 43)
	Polystyrene MPs (PS-MPs)	Rat	Inhalation	Inflammatory response	MPs increase the expression of inflammatory proteins (TGF-β and TNF-α) in rat lung tissue.	Lim et al. (119)
	Polystyrene MPs (PS-MPs)	Mouse	Inhalation	Inflammatory response	PS-MPs cause early lung tissue inflammation through the activation of the NLRP3/caspase-1/IL-1β signaling pathway.	Wu et al. (120)

### 6.1 Neurotoxicity: damage to the blood–brain barrier

MPs have the capability to traverse the blood–brain barrier and accumulate within brain tissue. Research indicates that smaller MPs are more likely to penetrate this barrier. For instance, polystyrene microplastic particles (PS-MPs) measuring 50 nm can cross into the mouse brain, where they activate microglial cells and inflict significant neuronal damage (75). Additionally, PS-MPs have been shown to reduce levels of synaptic proteins, disrupt neurotransmitter function, induce neuroinflammation, and lead to deficits in learning and memory in mice (80–82). The neurotoxic effects of MPs are further evidenced by declines in motor abilities across certain species, which manifest as behavioral changes (83). This indicates that MPs could play a role in the onset of neurodegenerative disorders, such as Parkinson’s disease, Huntington’s disease, and Alzheimer’s disease (84–87).

### 6.2 Cardiopulmonary toxicity

Recent research has identified various types of MPs in different cardiac tissues, with the largest diameter recorded at 469 μm (88). The direct cardiotoxic effects of MPs encompass arrhythmias, compromised cardiac function, pericardial edema, and myocardial fibrosis. At the microvascular level, MPs can induce hemolysis, thrombosis, blood coagulation, and damage to vascular endothelium, primarily through mechanisms involving oxidative stress, inflammation, and apoptosis (89). In an *in vitro* study utilizing a three-dimensional cardiac organoid model derived from human pluripotent stem cells, exposure to MPs significantly altered the expression of genes associated with cardiac hypertrophy (MYH7B/ANP/BNP/COL1A1), while cardiac-specific markers such as MYL2, MYL4, and CX43 were also notably elevated (90). Additionally, in a zebrafish embryo model, exposure to MPs increased the incidence of thrombosis and exacerbated atherosclerosis by activating and upregulating macrophage receptors. Toxic effects of MPs on the cardiovascular system have also been documented in zebrafish, mouse, and chicken models (Table 1) (91, 92).

### 6.3 Hepatotoxicity

The gastrointestinal tract serves as the primary entry point for MPs. Research has demonstrated that the accumulation of MPs in the intestines can activate immune and inflammatory responses in the intestinal mucosa, potentially leading to damage to the mucosal lining (93). Studies indicate that once MPs enter the intestines, they can trigger inflammatory reactions in murine models (94, 95). Additionally, a series of *in vitro* investigations have revealed that exposure to MPs can result in oxidative stress, DNA damage, inflammatory responses, cell membrane injury, and apoptosis of intestinal epithelial cells, ultimately compromising intestinal barrier function (96–98).

Given that the liver is the main organ responsible for detoxifying exogenous substances, the effects of MPs on liver function are varied. Research involving mice has shown that polystyrene microplastics (PS-MPs) accumulate in the liver and contribute to insulin resistance by promoting inflammation and inhibiting insulin signaling pathways (99). In zebrafish, MPs have been found to induce lipid accumulation and potentially cause histological damage, including necrosis and hemorrhage (100). In mice, liver damage from PS-MPs is indicated by elevated levels of alkaline phosphatase (ALP) aspartate, alanine aminotransferase (ALT), aminotransferase (AST), and lactate dehydrogenase (LDH), alongside hepatotoxicity and dysbiosis of gut microbiota (101). Notably, one study reported that PS-MPs exacerbated lipid metabolism abnormalities in diabetic mice, leading to heightened inflammatory responses. This suggests that individuals with chronic conditions may exhibit increased sensitivity to plastic pollution (102).

### 6.4 Respiratory toxicity

Numerous studies have indicated that inhalation of MPs present in the atmosphere can lead to significant pulmonary toxicity and respiratory conditions, including asthma, pneumonia, emphysema, and allergic rhinitis. Animal research has consistently demonstrated that inhaling MPs can trigger pulmonary inflammatory responses, intensify oxidative stress, and even result in pulmonary fibrosis (103, 104). For instance, studies involving the intratracheal administration of amino-polystyrene nanoplastics (APS-NPs) in mice have revealed inflammatory infiltration in lung tissue (105). Moreover, a variety of in vitro investigations have shown that MPs can induce oxidative stress, inflammatory responses, genetic damage, and apoptosis in lung cells (32, 33, 42, 43, 106, 107). Recent findings also suggest that respiratory Ingestion of PS-MPs can disrupt the balance of nasal and pulmonary microbiota (108). Additionally, a clinical cohort study identified a higher concentration of MPs in patients diagnosed with allergic rhinitis (109).

## 7 Hazards of MPs interacting with other pollutants

MPs have a significant surface area and exhibit hydrophobic properties, allowing them to function as carriers for various environmental pollutants, such as organic materials, heavy metals, and pathogenic microorganisms. This adsorption can result in exacerbated effects on biological organisms and human health (1).

### 7.1 MPs interact with organic matter

The interplay between MPs and environmental organic compounds can facilitate bioaccumulation and intensify toxic effects in living organisms. For instance, the interaction between F-53B and PS-MPs significantly increased the transcription of pro-inflammatory genes such as cxcl-clc and il-1β, while also elevating the levels of inducible nitric oxide synthase (iNOS) protein in zebrafish larvae, thereby inducing inflammatory stress in these fry (110). Additionally, another study demonstrated that the combination of MPs with polybrominated diphenyl ethers (PBDEs) worsened developmental and thyroid toxicity in zebrafish (81, 82).

### 7.2 MPs interact with heavy metal

MPs can serve as carriers for various environmental heavy metals, such as arsenic (As), cadmium (Cd), and lead (Pb). When these MPs, laden with heavy metals, are introduced into organisms or the human body, they can pose significant health risks. Research involving oysters demonstrated that the presence of MPs combined with arsenic resulted in synergistic effects on gene expression, which in turn led to heightened oxidative stress and increased apoptosis (34, 111).

### 7.3 MPs interact with causative agent

MPs can also carry potential pathogenic microorganisms, leading to human infections with pathogens such as pathogenic Vibrio species (112). A study has shown that MPs can carry the novel coronavirus, potentially accelerating its spread and infection (113).

In summary, as a carrier of environmental pollutants, microplastics can amplify their health risks by adsorbing organic pollutants, heavy metals and pathogens. This further highlights the need to study the synergistic toxicity of microplastics.

## 8 Conclusion

The pervasive presence of MPs in various environmental matrices poses significant challenges to public health, necessitating urgent attention from the scientific community and policymakers alike. This review elucidates the complex interactions between MPs and human health, revealing their potential to disrupt cellular functions, induce inflammatory pathways, and exacerbate oxidative stress. The evidence presented underscores the multifactorial nature of MP toxicity, particularly when considered alongside other environmental contaminants, which may synergistically enhance health risks.

Moving forward, it is imperative to adopt a multidisciplinary approach to further investigate the biological mechanisms underlying MP-induced toxicity. Future research should prioritize longitudinal studies that assess the long-term health impacts of chronic MP exposure, as well as the identification of vulnerable populations who may be disproportionately affected. Additionally, the development of standardized methodologies for the detection and quantification of MPs in biological samples will be crucial for advancing our understanding of their bioaccumulation and systemic effects.

To address the escalating health risks posed by microplastics (MPs), a multi-tiered strategy integrating policy, research, and technological innovation is imperative. Governments must strengthen regulations to phase out single-use plastics and microbeads while mandating the adoption of advanced filtration technologies in wastewater treatment systems to intercept MPs. Concurrently, research priorities should focus on establishing standardized toxicity assessment protocols, investigating long-term bioaccumulation effects, and elucidating the synergistic interactions between MPs and co-pollutants (e.g., heavy metals, pathogens), with targeted studies on vulnerable populations. Technological advancements in biodegradable materials and enhanced recycling methodologies are critical to reducing plastic dependency. Furthermore, public awareness campaigns should promote behavioral modifications, such as minimizing bottled water consumption and prioritizing natural textiles. These measures aim to mitigate the environmental and health threats posed by MPs and drive societal transitions toward sustainability. This comprehensive approach not only addresses current challenges but also provides a framework for future research and practice to foster a greener and healthier ecological environment. Ultimately, addressing the public health implications of microplastics requires collaborative efforts across disciplines, including toxicology, epidemiology, and environmental science. By fostering a comprehensive understanding of MPs’ health impacts, we can inform regulatory frameworks and public health strategies aimed at reducing exposure and mitigating the risks associated with this emerging environmental threat. The urgency of this issue calls for immediate action to safeguard human health and preserve the integrity of our ecosystems.

## Footnotes

^1^ https://plasticseurope.org
